# Phosphorylation of the Arp2 Subunit Relieves Auto-inhibitory Interactions for Arp2/3 Complex Activation

**DOI:** 10.1371/journal.pcbi.1002226

**Published:** 2011-11-10

**Authors:** Arjun Narayanan, Lawrence L. LeClaire, Diane L. Barber, Matthew P. Jacobson

**Affiliations:** 1 Graduate Group in Biophysics, University of California, San Francisco, San Francisco, California, United States of America; 2 Department of Cell and Tissue Biology, University of California, San Francisco, San Francisco, California, United States of America; 3 Department of Pharmaceutical Chemistry, University of California, San Francisco, San Francisco, California, United States of America; University of Michigan, United States of America

## Abstract

Actin filament assembly by the actin-related protein (Arp) 2/3 complex is necessary to build many cellular structures, including lamellipodia at the leading edge of motile cells and phagocytic cups, and to move endosomes and intracellular pathogens. The crucial role of the Arp2/3 complex in cellular processes requires precise spatiotemporal regulation of its activity. While binding of nucleation-promoting factors (NPFs) has long been considered essential to Arp2/3 complex activity, we recently showed that phosphorylation of the Arp2 subunit is also necessary for Arp2/3 complex activation. Using molecular dynamics simulations and biochemical assays with recombinant Arp2/3 complex, we now show how phosphorylation of Arp2 induces conformational changes permitting activation. The simulations suggest that phosphorylation causes reorientation of Arp2 relative to Arp3 by destabilizing a network of salt-bridge interactions at the interface of the Arp2, Arp3, and ARPC4 subunits. Simulations also suggest a gain-of-function ARPC4 mutant that we show experimentally to have substantial activity in the absence of NPFs. We propose a model in which a network of auto-inhibitory salt-bridge interactions holds the Arp2 subunit in an inactive orientation. These auto-inhibitory interactions are destabilized upon phosphorylation of Arp2, allowing Arp2 to reorient to an activation-competent state.

## Author Summary

The Arp2/3 complex consists of seven associated protein subunits including Arp2 and Arp3 that play a central role in the formation of actin filaments. Filament formation by the Arp2/3 complex drives important cell processes such as cell movement and endocytosis. The function of the Arp2/3 complex is highly regulated, and improper regulation of its activity has been linked to cancer metastasis. One level of regulation is post-translational phosphorylation, in which a −2 charged phosphate group is added to the uncharged amino acids threonine 237 and 238 of Arp2. We use molecular dynamics simulations and biochemical studies to show that Arp2 phosphorylation results in large structural changes of the Arp2/3 complex consistent with low-resolution structural studies. The simulations suggest phosphorylation allows the complex to reorient to an activation competent state by destabilizing interactions that hold Arp2 in an inactive position. Further simulations suggested that mutation of the Arp2/3 complex could allow complex activation, and we verified this gain-of-function mutation biochemically. We propose a model for Arp2/3 complex activation in which phosphorylation destabilizes the inactive state of the complex, allowing structural changes that are permissive for activation by nucleation-promoting factors and binding to the mother filament.

## Introduction

Spatial and temporal control of the assembly and disassembly of actin filaments is crucial for a number of distinct cell processes, including endocytosis and cell migration [1]. The spontaneous assembly of actin filaments from a pool of actin monomers requires the formation of an unstable actin trimer nucleus, from which further polymerization is thermodynamically favorable [2]. Fast filament assembly is achieved by several classes of proteins that act as actin nucleators, constituting one level of regulation. Formins and the spire proteins nucleate unbranched filaments [3], [4] and the Arp2/3 complex facilitates assembly of branched filaments by nucleating a new “daughter” filament from the side of an existing “mother” filament [5], [6]. Branched filament networks generated by the Arp2/3 complex are required to build many cellular structures, and Arp2/3 complex is the primary nucleator of new actin filaments in most crawling cells (reviewed in [1], [7], [8]). Aberrant Arp2/3 complex function has been implicated in a number of disease conditions, most notably cancer metastasis [7], [9].

Direct regulation of the nucleating activity of the Arp2/3 complex leads to a second level of control over actin filament assembly. The Arp2/3 complex is composed of seven subunits: the actin-related proteins Arp2 and Arp3, and ARPC1–5. While binding of the mother filament contributes significantly to activation [10], full activity of the Arp2/3 complex also requires ATP binding to Arp2 and Arp3 [11], [12] and binding of a nucleation promoting factor (NPF), such as WASP [13], [14], N-WASP [15], SCAR/WAVE [16], [17], and the pathogenic proteins ActA from *Listeria monocytogenes* [18] and RickA from *Rickettsia* [19], [20]. NPF binding to actin monomers facilitates the nucleation reaction, and NPFs couple Arp2/3 complex activity to that of Rho-family GTPases [21], [22].

The structures of the *apo* and nucleotide-bound states of Arp2/3 were revealed by X-ray crystallography [23], [24], [25], and these lead to the hypothesis that activation required large structural changes [25]. The structure of the active Arp2/3 complex at the junction of the mother filament and the newly nucleated daughter filament (the branch junction) was recently revealed in reconstructions from electron micrographs of negatively-stained specimens [26], [27]. In support of the hypothesized structural changes, docking of the inactive Arp2/3 complex crystal structure into the branch junction density revealed substantial rearrangements of subunits, particularly of the Arp2 and ARPC3 subunits [27]. For the Arp2/3 complex to incorporate into the daughter filament and increase filament assembly, Arp3 and Arp2 appear to undergo a large change in their relative orientation from their arrangement in the inactive crystal structure to their conformation in the branch junction density, in which they appear to mimic the short-pitch of an actin dimer [25], [27].

An additional, more recently identified requirement for activating nucleation by the Arp2/3 complex is threonine or tyrosine phosphorylation of the Arp2 subunit [28]. Mass spectrometry of purified Arp2/3 complex revealed phosphorylation of Arp2 T237 and T238. Although no phosphorylated tyrosine was identified by mass spectrometry, mutagenesis studies suggested Arp2 Y202 as the likely phosphorylation site. Mapping of the Arp2 phosphorylation sites onto the crystal structure reveals that these residues are near the interface of Arp2, Arp3, and ARPC4, and we predicted that phosphorylation of these residues could play a role in the large conformational changes predicted upon activation [28]. Consistent with this prediction, our biochemical assays suggested that Arp2 phosphorylation primes the complex for activation to allow conformational changes predicted to be necessary for activation [28]. However, the mechanism by which phosphorylation permits activation of the Arp2/3 complex remains poorly understood.

Computational studies have the potential to elucidate aspects of Arp2/3 complex function and regulation. While the impact of computation in this regard has been limited thus far due to the large system size, molecular dynamics simulations have been used to examine dynamics of the ATP binding cleft in Arp2 and Arp3 [29], [30]. In addition, homology modeling of the structures of the Arp2/3 complex from different species has generated hypotheses about functionally important surfaces [31]. Recently, steered molecular dynamics simulations were used to investigate potential pathways of Arp2/3 complex in the absence of phosphorylation [32], and molecular dynamics and protein-protein docking was used to generate a model of mother filament bound to the Arp2/3 complex, which was then validated experimentally [33]. Computational methods, molecular dynamics methods in particular, have also been used previously to study conformational changes of other proteins upon phosphorylation (reviewed in ref. [34]). Examples include the study of structural changes caused by phosphorylation in the activation and glycine-rich loops of protein kinases [35], [36], [37], [38], changes in peptide conformations [39], [40], and in membrane proteins such as phospholamban [41].

Here, we use unbiased molecular dynamics simulations to determine how phosphorylation at Arp2 T237 and T238 may change the structure of the Arp2/3 complex and permit activation by NPFs. We find large conformational changes in the Arp2/3 complex upon phosphorylation, including the reorientation of Arp2 relative to Arp3, toward the short-pitch dimer orientation. Our simulations suggest a mechanism by which a complex network of positively and negatively charged amino acids at the Arp2/Arp3/ARPC4 interface holds the complex in an inactive configuration, and phosphorylation disrupts these auto-inhibitory interactions. To test this prediction, we designed, based on further computational simulations, mutations of the Arp2/3 complex that we predicted would disrupt the auto-inhibitory interactions. Biochemical assays reveal that this mutant, R105/106A ARPC4, does in fact show nucleation activity even in the absence of NPFs.

## Results

### Phosphorylation of Arp2 at T237/238 or Y202 is required for full Arp2/3 complex nucleation activity

**Figure 1:**
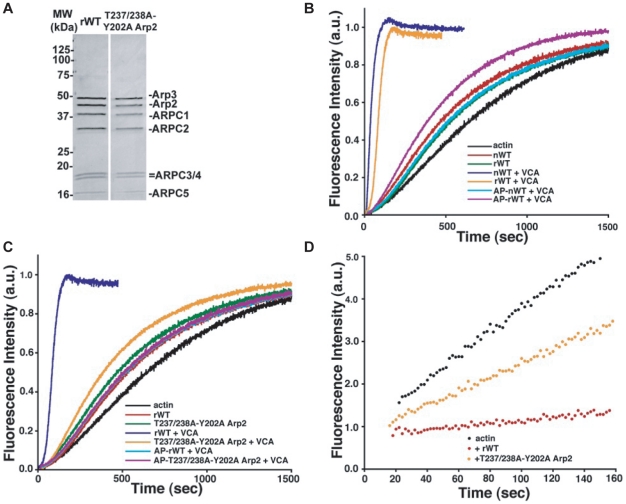
Phosphorylation site mutant Arp2/3 complex (T237/238A-Y202A Arp2) nucleates actin filaments less efficiently than recombinant WT.

We previously reported that phosphorylation of Arp2 T237/238 or Y202 is necessary for activation of the Arp2/3 complex in the presence of NPF [28]. Phosphorylation of T237/238 in endogenous Arp2 was confirmed by mass spectrometry, and heterologous expression of Arp2 with alanine substitutions in T237/238 and Y202 inhibits membrane protrusion. We tested nucleation activity of a mutant Arp2/3 with these residues mutated to alanine using recombinant Arp2/3 complex generated in a baculovirus expression system and purified as previously described [42]. We mutated all three residues because we previously showed that phosphorylation of these sites acts as a logical ‘or gate’ with either being necessary for activation [28]. Subunits of the wild type (WT) and mutant Ala-substituted T237/238-Y202 Arp2 (T237/238A-Y202A) Arp2/3 complex were expressed independently in *Spodoptera frugiperda*s (Sf21) insect cells, and were confirmed to assemble the seven-subunit complex with equimolar stoichiometry (**Fig. 1a**). We also confirmed that binding to NPF (N-WASP-VCA) (K_d_ = 0.5 µM) and actin filaments (K_d_ = 1.2–1.3 µM) is similar for WT and T237/238A-Y202A Arp2 rArp2/3 complex (**Fig. S1a,b**).

The rate of assembly of pyrene-labeled actin into filaments, an index of Arp2/3 complex nucleation activity, was similar with WT rArp2/3 (0.193 nM filament ends) and with Arp2/3 complex purified from bovine thymus (0.209 nM filament ends at concentrations of 5 nM Arp2/3 complex with 4 µM actin) (**Fig. 1b** and **Fig. S2a**). In the presence of NPF (C-terminal VCA domain of N-WASP), assembly rates increased 19-fold and were 3.30 nM and 2.51 nM filament ends for recombinant and native Arp2/3 complex respectively (**Fig. S2a**). We previously showed that native Arp2/3 complex from bovine thymus and WT rArp2/3 purified from insect cells are phosphorylated, and that native Arp2/3 complex pretreated with the dual specificity alkaline phosphatase Antarctic phosphatase (AP) is not activated by NPFs [28]. We confirmed that nucleation by native Arp2/3 and WT rArp2/3 complex in the presence of NPFs was reduced after treatment with AP to levels similar to untreated rArp2/3 complex in the absence of NPF (**Fig. 1b** **and Fig. S2a**). These data indicate that the activity of WT rArp2/3 in the absence and presence of NPF is similar to that of native Arp2/3 complex and that dephosphorylation inhibits NPF-induced activity.

In the absence of NPF, the rate of actin filament assembly and the concentration of filament ends with mutant rArp2/3 complex containing T237/238A-Y202A Arp2 (t_1/2_ = 500 s, 0.260 nM) were similar to native and WT complexes (**Fig. 1c** **and Fig. S2b**). In the presence of NPF, although the rate of filament assembly with the mutant decreased to t_1/2_ = 348 s, it was 4-fold slower than the rate of t_1/2_ = 84 s with WT rArp2/3 and similar to that of NPF-stimulated WT rArp2/3 complex pretreated with AP. The concentration of filament ends for AP-treated Arp2/3 complex and the mutant was similar in samples with or without NPF (**Fig. S2a and S2b**). Pretreating the mutant with AP completely blocked the increased rate of filament assembly in the presence of NPF, and rates were similar to WT and mutant in the absence of NPF. These findings indicate that phosphorylation of T237/238 or Y202 of the Arp2 subunit is necessary for maximal nucleation activity of the Arp2/3 complex. However, mutant rArp2/3 complex containing T237/238A-Y202A Arp2 retains some residual activity that is abolished by AP.

To nucleate a new actin filament, the Arp2/3 complex binds to filament pointed ends, which reflects its capping activity. We used actin seeds capped at the barbed end with gelsolin to measure pointed end capping by WT and mutant Arp2 rArp2/3 complex. Gelsolin-capped actin filaments elongated from their pointed ends in the presence of 4 µm actin (**Fig. 1d**). Addition of WT rArp2/3 complex slowed filament assembly from the pointed end. The rArp2/3 complex containing mutant Arp2 also slowed filament assembly, although markedly less than with WT. The measured affinity of the mutant for the pointed end decreased approximately 8-fold (**Table S1**). These data suggest that phosphorylation of T237/238 or Y202 in Arp2 is necessary for rArp2/3 complex to efficiently bind the pointed end of actin filaments.

### Computational simulations reveal that phosphorylation induces large conformational changes in the Arp2/3 complex

**Figure 2:**
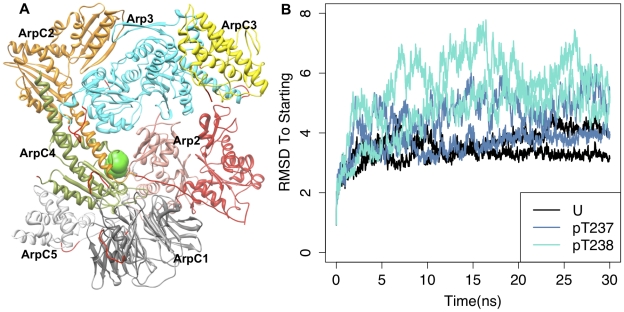
Arp2 phosphorylation induces structural changes in simulations of wild-type Arp2/3 complex.

We previously hypothesized that phosphorylation may induce a conformational change that allows activation by mother filament and NPF [28]. To test this hypothesis, we performed molecular dynamics simulations on phosphorylated and unphosphorylated wild-type Arp2/3 complex, using the inactive conformation observed in the crystal structure [25] as a starting point (**Fig. 2a**). We reasoned that, if our hypothesis were correct, the unphosphorylated wild-type structure should remain relatively unperturbed during the molecular dynamics simulation, while phosphorylated Arp2/3 should show conformational changes caused by the strong electrostatic perturbation associated with introducing the phosphate groups.

Due to the large size of the Arp2/3 complex, the simulations required substantial computational resources on a supercomputer. We performed simulations only with Arp2 T237 and T238 individually phosphorylated, as well as the unphosphorylated ‘control’ simulation. For each of these systems, we ran duplicate 30 ns molecular dynamics simulations to control for simulation dependence on the initial conditions and stochastic fluctuations. Observing large conformational changes using molecular dynamics simulations is difficult because of the gap between experimental and computationally feasible timescales, and we do not expect to see the full range of structural change in these simulations. Nonetheless, large conformational changes were observed for Arp2/3 when phosphorylated on either T237 or T238 of Arp2. Backbone root mean square deviations (RMSDs) following global alignment of simulation snapshots to the starting model revealed modest conformational changes of 3–4 Å RMSD for the unphosphorylated simulations compared with larger conformational changes of 4–8 Å for the phosphorylated simulations (**Fig. 2b**). In general, the directionality of the conformational changes relative to the unphosphorylated simulations with pT237 (phosphorylated T237) or pT238 were qualitatively similar, as were the results in the two duplicate simulations for each system (**Fig. S3b**). The conclusions we draw are supported by all of the simulations, although the precise details of the dynamical behaviors differed. The unphosphorylated simulations show convergence over the last 20 ns of simulation time. Therefore, the last 20 ns were used in the analyses below. This convergence with regard to simulation time does not indicate equilibrium convergence; it is possible and even likely that the full range of structural changes in the phosphorylated simulation in particular have not been realized (see Discussion). It should also be noted that these structural changes were not due to artifactual steric effects of adding phosphate groups to construct the phosphothreonine side-chains. The phosphate groups were added without causing any steric clashes with surrounding residues (data not shown). The starting structure of Arp2 subdomains 1 and 2, which are disordered in all but one crystal structure, which was stabilized with glutaraldehyde [24], were homology modeled based on the actin monomer structure (PDB 1ATN [43]).

**Figure 3:**
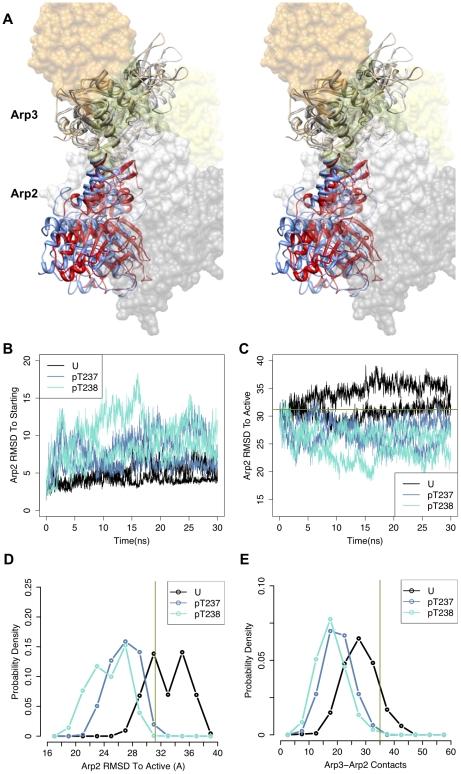
Changes in Arp2-Arp3 orientation upon phosphorylation. (a) Arp3 and Arp2 subunits from snapshots from the last ns of the unphosphorylated (Arp3-gray; Arp2-blue) and phosphorylated T237 Arp2 (tan, red) wild-type simulations are shown following alignment of Cα atoms of subdomains 1 and 2 of Arp3. These and all other structural figures were produced using the molecular graphics program UCSF Chimera [56], except where indicated. The other subunits of the complex from the snapshot of the unphosphorylated simulation, colored as in Fig. 2a, were represented as transparent surfaces in order not to occlude the views of Arp2 and Arp3. (b) RMSD of Arp2 Cα atoms to their initial positions vs. simulation time following alignment of subdomains 1 and 2 of Arp3. (c) RMSD of Arp2 Cα atoms to their positions in the model of the active short-pitch dimer orientation (B. Nolen, personal communication) vs. simulation time following alignment of subdomains 1 and 2 of Arp3. (d) Distribution of root-mean-square deviations (RMSD) of Cα atoms of Arp2 from the MD trajectory to Arp2 atoms in the active short-pitch dimer orientation after alignment of subdomains 1 and 2 of Arp3 over the last 20 ns of simulations. (e) Distribution of number of contacts between Arp3 and Arp2 heavy atoms over the last 20 ns of simulations. In (b)–(e), coloring is as follows: U(black)- unphosphorylated; pT237(blue-gray) – phosphorylated T237 Arp2; pT238(cyan) – phosphorylated T238 Arp2. RMSD of Arp2 Cα atoms in the initial model to their positions in the active orientation is 31.2 Å, and the number of Arp3-Arp2 contacts in the initial model is 35. These values are indicated for reference in the appropriate plots with a green line.

The conformational changes induced by phosphorylation were dominated by changes in the orientation of Arp2, ARPC1, and ARPC3 relative to other subunits (**Fig. S3**). In particular, phosphorylation induced motion of the Arp2 subunit relative to the Arp3 subunit toward its active position as a mimic of an actin short-pitch dimer [27], a conformation required for polymerization of actin (**Fig. 3A** and **Fig. S4**). To quantify this motion, we used the model of active Arp2/3 obtained by orienting Arp2 and Arp3 as in an actin short-pitch dimer, with no changes in the structure or orientation of other subunits [B. Nolen, personal communication]. Specifically, we computed the Cα root mean square deviation (RMSD) of the Arp2 subunit between individual snapshots over the last 20 ns and Arp2 in the starting (**Fig. 3b**) or active orientation (**Fig. 3c**) with respect to Arp3 following alignment of experimentally resolved Cα atoms of Arp3 subdomains 1 and 2. Phosphorylation of either T237 or T238 caused conformational changes away from the starting inactive state relative to that observed in the simulation of unphosphorylated Arp2/3. Phosphorylation also appears to cause conformational changes that lower the RMSD of the Arp2 subunit to the active orientation, although further large conformational changes are required for full activation (**Fig. 3d**). This may be in part to the relatively short timescale of the simulations. However, phosphorylation is not expected to induce a conformational change to the fully active state because our biochemical determinations (**Fig. 1b** and [28]) indicate phosphorylation is necessary but not sufficient for full activation, which also requires binding of the mother filament and NPFs.

Analyzing the Cα RMSD of each residue of Arp2 after alignment of Arp3 subdomains 1 and 2 reveals that the largest changes in Cα position of Arp2 occur in the C-terminal tail as well as in subdomains 1 and 2, which are disordered in most unphosphorylated Arp2/3 complex crystal structures (**Fig. S5a**) [23], [24], [25]. Larger structural changes are induced in the phosphorylated simulations than in the unphosphorylated simulations across the entirety of Arp2. Substantial increases in the Arp2 per-residue RMSD after alignment of Arp3 subdomains 1 and 2 compared with those after alignment of the Arp2 Cα atoms suggest that the motion of Arp2 largely consists of a rigid-body movement (**Fig. S5b**). Phosphorylation induces the loss of contacts between Arp2 and Arp3 subunits, potentially allowing the reorientation of these subunits (**Fig. 2e**).

**Figure 4:**
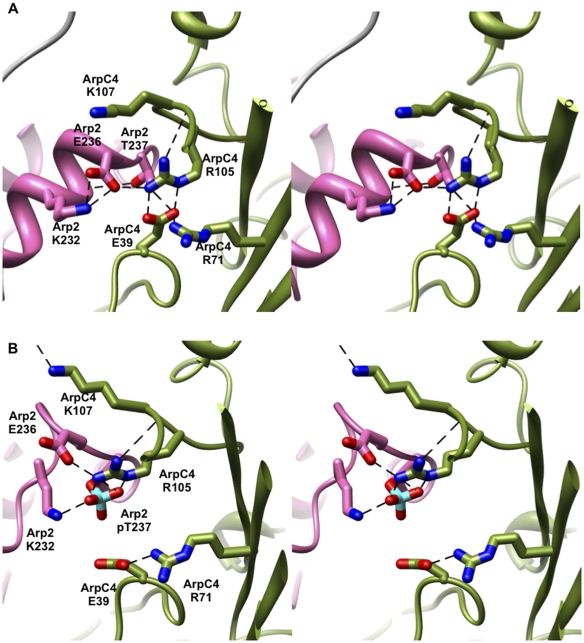
Interactions in the vicinity of the T237 Arp2 phosphorylation site. Stereoimages of the salt-bridge network between Arp2 (pink) and ARPC4 (green) in simulations of wild-type (a) unphosphorylated or (b) phosphorylated Arp2 T237 Arp2/3 complex.

The results of the molecular dynamics simulations are consistent with the hypothesis that phosphorylation induces conformational changes that contribute to adopting a nucleation-competent form [28]. We examined the vicinity of the phosphorylation sites in detail to identify the interactions mediating these structural changes. In the unphosphorylated state, T237 and T238 are located near the interface between Arp2 and ARPC4, and are also close to the interfaces with Arp3 and ARPC2. The interactions between Arp2 and ARPC4 in the vicinity of T237/238 are dominated by salt bridges, several of which are highlighted in **Fig. 4a** and **Fig. S6a**. In particular, the complex electrostatic network involves E39, R71, E99, R105, R106, and K107 on ARPC4; E236 and K232 on Arp2; and R409 and E121 on Arp3. Unphosphorylated T237 and T238 do not participate in the side-chain hydrogen-bonding network.

By contrast, these interactions were dramatically rearranged with phosphorylation of either T237 or T238 (**Fig. 4b** and **Fig. S6b**). LeClaire, et al. hypothesized that R105 and R106 of ARPC4 mediate the effects of phosphorylation at T237 and T238 of Arp2, respectively [28]. The molecular dynamics simulations support this hypothesis but also suggest a much more complex rearrangement of the electrostatic network driven by introduction of the phosphate charge. The strengths of salt bridge interactions between phosphorylated amino acids and lysine/arginine side chains are stronger than those between aspartate/glutamate and lysine/arginine, with phosphate-arginine interactions being particularly stable [44]. Thus, unsurprisingly, the phosphorylated amino acids form both transient and stable interactions with arginine residues in the simulations, such as between pT237 of Arp2 and R105 of ARPC4 (**Fig. 4b**) and between pT238 of Arp2 and R106 of ARPC4 (**Fig. S6b**). The incorporation of pT237 and pT238 into the electrostatic network necessitates that other salt bridging interactions are disrupted and a new set of interactions are formed, either as a direct consequence or as an indirect result of the induced conformational changes (**Fig. S7**).

Because T237 and T238 are near the interfaces with several other subunits, perturbations to the electrostatic network induced by phosphorylation can cause large conformational changes. We hypothesized that mutating key residues that interact with pT237 and pT238 would abolish the ability of phosphorylation to induce these conformational changes, and hence activation of the nucleation activity. This hypothesis is based on studies of phosphorylation-mediated activation in systems such as protein kinases, in which attractive interactions with arginine residues that interact with phosphorylated residues drive conformational changes key to phospho-activation [45]. In particular, the simulations predicted that R105 of ARPC4 would form a specific and stable ion pair with the phosphate on T237 of Arp2. To test this hypothesis, we constructed R105A ARPC4 mutants with unphosphorylated and phosphorylated T237 *in silico*, and generated two independent 30 ns molecular dynamics simulations for each.

**Figure 5:**
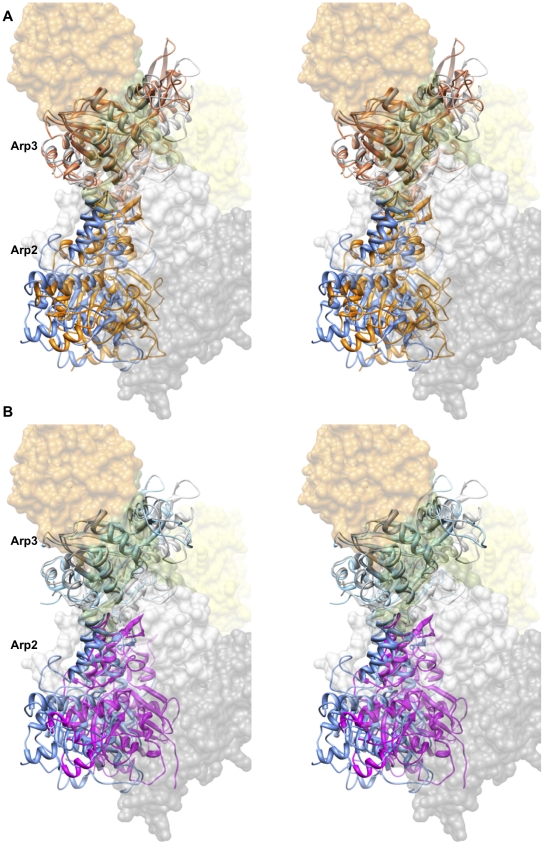
Structural changes induced by R105A ARPC4 mutants in the absence and presence of phosphorylation. Stereoimages of changes in Arp2-Arp3 orientation in R105A ARPC4 mutant Arp2/3 complex from snapshots from the last ns of the unphosphorylated wild-type(Arp3-gray; Arp2-blue) and the (a) unphosphorylated R105A ARPC4 mutant complex (pink, orange) or (b) phosphorylated T237 Arp2 ARPC4 R105A ARPC4 mutant complex (cyan, magenta) simulations are shown following alignment of Cα atoms of subdomains 1 and 2 of Arp3. The other subunits of the complex from the snapshot of the unphosphorylated simulation, colored as in Fig. 2a, were represented as transparent surfaces in order not to occlude the views of Arp2 and Arp3.

**Figure 6:**
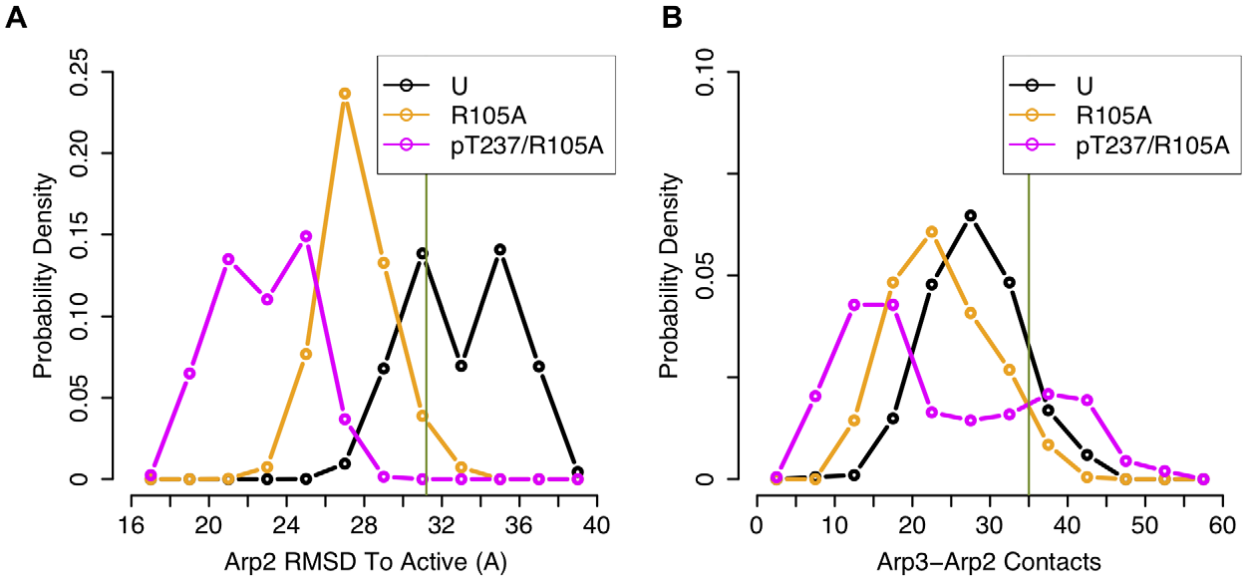
R105A ARPC4 mutant complexes alter Arp2-Arp3 orientation and contacts. (a) Distribution of root-mean-square deviations (RMSD) of Cα atoms of Arp2 to Arp2 atoms in the active short-pitch dimer orientation (B. Nolen, personal communication) after alignment of subdomains 1 and 2 of Arp3 over the last 20 ns of simulations. (b) Distribution of number of contacts between Arp3 and Arp2 heavy atoms over the last 20 ns of simulations. Coloring is as follows: U(black) – unphosphorylated; R105A(orange) – unphosphorylated R105A ARPC4 mutant; and pT237/R105A(magenta) – Arp2 T237 phosphorylated R105A ARPC4 mutant. Green line represents the corresponding RMSD (31.2 Å) and number of contacts (35) for the unphosphorylated starting model as in Fig. 3.

Contrary to our expectations, simulations of the unphosphorylated Arp2/3 complex with the R105A ARPC4 mutation produced a similar, but somewhat smaller, structural change to that induced by phosphorylation at T237 or T238 Arp2 (**Fig. 5a** and **6**). This result suggested that the R105A ARPC4 mutation could allow partial activation of Arp2/3 even in the absence of phosphorylation. Phosphorylation of T237 Arp2 in the context of the R105A ARPC4 mutant produced even larger structural changes than phosphorylation of T237 or T238 Arp2 alone (**Fig. 5b** and **6**). It should again be noted that the structures at the end of these simulations likely do not represent the full range of conformational change of these complexes due to the short timescales available to MD simulation. However, the large conformational changes away from the inactive, initial state observed at these short timescales are similar to the conformational changes caused by phosphorylation, suggesting increased activity of these mutants.

### rArp2/3 complex with R105/106A ARPC4 is active in the absence of an NPF

**Figure 7:**
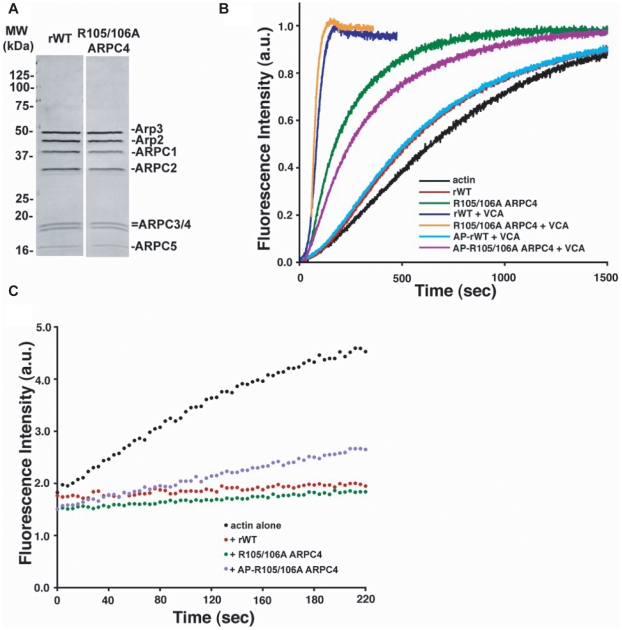
R105/106A ARPC4 mutant nucleates actin filaments without nucleation promoting factors. (a) Recombinant Arp2/3 complex WT (rWT) and R105/106A ARPC4 expressed and purified from sf21 cells. (b) Pyrene actin assembly assays comparing nucleation activity of rWT and R105/106A ARPC4 rArp2/3 complex. (c) Pointed end capping assay with actin alone (black), rWT (red) and R105/106A ARPC4 rArp2/3 (green), and R105/106A ARPC4 rArp2/3 pretreated with Antarctic phosphatase (AP-R105/106A ARPC4) (purple).

To test predictions from our molecular dynamics simulations on the role of arginines in ARPC4, we generated rArp2/3 complex with R105 and R106 of ARPC4 mutated to alanine (R105/106A ARPC4). While we did not simulate structural changes associated with the R106A ARPC4 mutation, the simulations indicated that R106 ARPC4 upon T238 phosphorylation played a role analogous to that of R105 ARPC4, forming a stable interaction with the phosphate group (**Fig. S6b**). The mutant R105/106A ARPC4 rArp2/3 complex purified from Sf21 cells showed subunit stoichiometry (**Fig. 7a**), binding to NPF (K_d_ = 0.06 µM), and binding to actin filaments (K_d_ = 1.5 µM) (**Fig. S1**) similar to WT. In the absence of NPF, the rate of actin filament assembly was markedly faster with rArp2/3 complex containing R105/106A ARPC4 (t_1/2_ = 173 s) than with WT (t_1/2_ = 539 s) (**Fig. 7b**) and there was 2.5-fold more filament ends with 4 µM actin and 5 nM Arp2/3 complex (**Fig. S8**), indicating that the mutant is constitutively more active. In the presence of NPF, the rate of assembly for the mutant (t_1/2_ = 71 s) and WT (t_1/2_ = 84 s) was similar, as was the number of filament ends (**Fig. S8**), revealing that the mutation has no effect on maximal NPF-induced Arp2/3 complex activity. Preincubating the mutant with AP decreased actin nucleation rates but only slightly (**Fig. 7b**), and much less than with AP treatment of WT complex. These data and our molecular dynamics simulations suggest that mutating R105/106 disrupts auto-inhibitory interactions and releases the Arp2/3 complex structure to a conformation that is permissive for nucleation activity.

Pointed end capping by the R105/106A ARPC4 rArp2/3 complex appeared similar to WT rArp2/3. Using gelsolin-capped actin seeds, the rate of pointed end elongation was not significantly different between mutant and wild-type Arp2/3 complex, and treating with AP only slightly attenuated capping activity of the mutant (**Fig. 7c** and **Table S1**). Hence, the R105/106A ARPC4 mutation likely causes structural differences similar to those seen in activated WT rArp2/3 complex, and phosphorylation of the R105/106A mutant enhances this effect, consistent with the simulations of the R105A ARPC4 mutant.

## Discussion

### Significance of conformational changes observed in simulations

Our simulations revealed large structural differences between the phosphorylated or mutant complexes and the unphosphorylated wild-type complex. These structural changes included the movement of Arp2 toward the active short-pitch dimer orientation relative to Arp3. However, the conformational changes observed in these short molecular dynamics simulations are much smaller than those assumed to occur upon full activation, and even with longer simulations we would not expect the phosphorylated complex to adopt the putative active conformation, since phosphorylation is necessary but not sufficient for activation. Rather, we propose a model in which phosphorylation destabilizes the inactive state, leading to conformational changes that relieve the auto-inhibition and thus are permissive for full activation by NPF binding.

It is impossible to say whether the actual structural differences in response to phosphorylation or mutation are realized at the end of these simulations, but this is very unlikely. Each of the duplicate simulations of the same state of the complex show differences, even the dual simulations of the unphosphorylated state (see, for example, the two peaks in the Arp2 RMSD to the active orientation (**Fig. 3d**)), indicating that equilibrium convergence has not been achieved even for the unphosphorylated, wild-type complex. Consequently, we believe the structures at the end of these simulations simply suggest that either phosphorylation or mutation induces large conformational changes that shift Arp2 towards the active short-pitch dimer orientation – they *are not* a prediction of the structure of the Arp2/3 complex upon phosphorylation or mutation.

Besides the changes in the Arp2-Arp3 orientation, large changes in the orientation of ARPC1 and ARPC3 relative to Arp3 were observed upon phosphorylation (**Fig. S3**). We can only speculate about the mechanism by which phosphorylation effects these changes due to the long distance of these subunits from the phosphorylation site. Similar to results from recent steered molecular dynamics simulations [32], the movement of ARPC3 appears to be linked to changes in the bilobal structure of Arp3, and the movement of ARPC1 appears to be linked to the change in orientation of Arp2. Unlike their simulations however, we do not observe large changes between ARPC2 and ARPC4 – these changes may appear at larger changes in Arp2 orientation than are observed in our simulations or on a longer timescale. The lack of large changes in ARPC2 and APRC4 are consistent however with the fact that mother filament binding is not increased relative to the unphosphorylated state as ARPC2 and ARPC4 appear to be the main sites of mother filament binding [33]. Additionally, while some contacts between Arp2 and Arp3 were lost, Arp2 was not observed to fully dissociate from Arp3 or ARPC4 (data not shown).

The findings here and previously [28] indicate that phosphorylation is required for activation of the Arp2/3 complex. However, crystal structures of the Arp2/3 complex from preparations found to have nucleating activity do not show phosphorylation of Arp2. We confirmed that phosphatase treatment rendered similar preparations to those used in crystallographic studies inactive (Fig. 1 and [28]). We confirmed that loss of activity was due to dephosphorylation and not phosphatase binding to the complex or ATP dephosphorylation of the subunits, and our findings suggest that several populations of Arp2/3 complex exist in our preparations (data not shown). It is possible that phosphorylated Arp2/3 complex may not form crystals due to differences in conformation.

### Relief of auto-inhibition by phospho-regulation

In all cases in which phosphorylation is required for functional activation, the unphosphorylated state can be considered auto-inhibited. A common mechanism for converting from the auto-inhibited to the activated state is one in which phosphorylation induces attractive interactions between the phosphorylated residue and other residues that are required for activating structural changes. This is seen in Ser/Thr protein kinases, and also in more traditionally auto-inhibited systems such as the phosphorylation of the tail of Tyr protein kinases (reviewed in [45]).

In contrast, mutation of arginine residues in the Arp2/3 complex enhances activation by phosphorylation (**Fig. 5** and **6**). This suggests a distinct mechanism to that discussed above. Structurally, these results suggested that the effects of phosphorylation are better understood as relieving an auto-inhibitory interaction through repulsive forces rather than driving conversion towards the active state via the formation of attractive interactions. The introduction of phosphate groups with a −2 charge disrupts the complex electrostatic network at the inter-subunit interfaces near the threonine phosphorylation sites that hold the complex in an inactive state. The destabilization of the interaction network driven by the need to accommodate the electrostatic perturbation leads to conformational changes that are permissive for full activation by NPF binding. Mutation of ARPC4 R105 and R106 constitutes an electrostatic perturbation that destabilizes the inter-subunit interfaces akin to phosphorylation. Combining the electrostatic perturbations of T237 or T238 phosphorylation and mutation of ARPC4 R105/R106 appears to lead to larger conformational changes (**Fig. 5** and **6**) and higher activity in the absence of NPF (**Fig. 7**). In addition, several other salt-bridge interactions are broken upon phosphorylation (Fig. S7). Other mutations, particularly of positively charged amino acids such as K232 Arp2, R409 Arp3, and R200 Arp2 that show strong interactions in the unphosphorylated states that are then broken upon phosphorylation, could constitute electrostatic perturbations that would also magnify the destabilizing effects of phosphorylation. Further studies will have to determine the extent to which the breakage of these other interactions can contribute to activation.

Based on the activation model laid forth by Dalhaimer and Pollard [32], the phosphorylation-induced relief-of-autoinhibition may provide a reduced energy barrier for conversion to the active state upon mother filament and NPF binding, although there may be thermodynamic effects such as stabilization of the active complex that our current data do not reveal. In this model, mutation of R105/R106 ARPC4 in the context of phosphorylation may further reduce the barrier to forming the fully active complex such that binding to mother filament even in the absence of NPF can still result in a substantial increase in the activation kinetics, though our data indicate that NPF binding further accelerates this process.

Due to the large computational expense of the simulations (∼500,000 cpu-hours during the course of this study), we were unable to perform all of the potentially informative simulations. For example, we have not investigated phosphorylation of Y202 on Arp2, which has been proposed as the site of tyrosine phosphorylation and is located close to T237/238 Arp2. The proximity of Y202 to the salt-bridge network around T238 leads us to speculate that its phosphorylation would exert its effects by a similar mechanism, but this hypothesis remains to be examined. We have also not investigated dual phosphorylation of both T237 and T238 Arp2. Nonetheless, despite these limitations and the short timescale probed by molecular dynamics, the simulations suggested a structural mechanism for phosphoregulation of Arp2/3 and predicted a gain-of-function mutation, which was confirmed experimentally. As such, this study provides an example of how computational simulations can be used to create testable models of regulatory phosphorylation, which is valuable when it is difficult to obtain direct, atomic-resolution structural information, as is often the case. Here, we have provided a new model for Arp2/3 regulation in which a network of electrostatic interactions helps to hold the complex in the inactive state, and this auto-inhibition must be relieved by phosphorylation to permit activation.

## Methods

### Generation of mutant subunits

Plasmids encoding Arp2/3 complex subunits were obtained from M. Welch (UC Berkeley) and were generated as described [42]. Site directed mutagenesis was performed using a QuikChange Mutagenesis kit (Agilent Technologies) using the appropriate template. Primers used for T237/238A Arp2 mutation: 5′ primer (GAGCAGAAACTGGCCTTAGAAGCCGCAGTATTAGTTGAATCTTATACACTCCC) 3′ primer (GGGAGTGTATAAGATTCAACTAATACTGCGGCTTCTAAGGCCAGTTTCTGCTC), primers for the Y202A Arp2 mutation 5′ primer (CAAGCTACTTCTGTTGCGAGGAGCCGCCTTCAACCACTCTGCTGATTTTGAAAC), 3′ primer (GTTTCAAAATCAGCAGAGTGGTTGAAGGCGGCTCCTCGCAACAGAAGTAGCTTG). Primers used for the R105/106A ARPC4 mutations: 5′ primer (GAGAACTTCTTTATCCTTGCAGCGAAGCCTGTGGAGGGG), 3′ primer (CTCTTGAAGAAATAGGAAACGTCGCTTCGGACACCTCCCC).

### Expression and purification of rArp2/3 complex

Recombinant Arp2/3 complex was expressed and purified as described [42]. Briefly, Sf21 cells at a density 1.0×10^6^ cells/ml were infected with baculoviruses containing cDNA encoding subunits of the Arp2/3 complex at equal infection units. Cells were grown in sf900 media in suspension for 48 hours and then harvested by a 10 min 1000×g centrifugation. Recombinant Arp2/3 complex was affinity purified on Talon resin (Clonetech), and fully assembled complex was collected after passage over a Superdex 200 FPLC gel filtration column.

### Actin polymerization

Pyrene actin polymerization assays were performed with 4 µM monomeric actin containing 5% pyrene-labeled actin in KMEI (50 mM KCl, 1 mM MgCl_2_, 1 mM EGTA, and 10 mM imidazole, pH 7), 2.5 to 50 nM Arp2/3 complex, and 500 nM N-WASP VCA domain. Measurements were made with an RF-5301PC spectrophotometer (Shimadzu) at 1 s intervals. Growing filament ends were calculated by determining the rate of actin assembly at 80% of polymerization and using the relationship **R = k_+_[A][E]** where **R** is the rate of actin assembly, **k_+_** is the association rate constant (10 µM^−1^⋅s^−1^), **[A]** is the concentration of monomeric actin and **[E]** is the concentration of growing filament ends as described previously [17]. The concentration of Arp2/3 complex was varied from 0 to 50 nM and the number of growing filaments calculated for each condition. Pointed elongation from gelsolin-capped actin filaments was measured as described previously [6]. Gelsolin-capped actin filaments (100 nM) were used for pointed end binding assays. F-actin binding assays and *in vitro* dephosphorylation of the Arp2/3 complex were performed as described previously [28].

### Quantification of Arp2/3 complex NPF and Actin binding constants

Binding constants of Arp2/3 complex for NPFs were determined by using GST-NWASP VCA covalently coupled to Activated CH-sepharose 4B (GE Healthcare, Piscataway, NJ). GST-NPF-coupled beads were added to mock-treated or Antarctic phosphatase-treated Arp2/3 complex and incubated at room temperature for 30 min. NPF-coupled beads were spun at 700× g for 5 min, the supernatant removed and beads resuspended in SDS-PAGE sample buffer. Coomasie-stained gels were scanned and quantified using a LabWorks imaging system and LabWorks Software (UVI, CA). The data were plotted and fitted using GraphPad Prism software (GraphPad Software, Inc., San Diego, CA). Binding constants for Arp2/3 complex for actin filaments were determined by actin co-sedimentation as described [46].

### Molecular dynamics simulations

Systems were prepared for molecular dynamics simulations starting from the crystal structure of the *apo* bovine Arp2/3 complex (PDB 1K8K [25]). A complete model of the unphosphorylated, wild-type bovine Arp2/3 complex was generated using the Protein Local Optimization Program (PLOP) [47], [48], [49] by building in all atoms missing in the electron density (except Arp2 subdomains 1 and 2). Subdomains 1 and 2 of Arp2 were modeled in based on homology to the actin monomer structure (PDB 1ATN [43]). The 15-residue unstructured extension at the end of ARPC2 was energy minimized, as were residues 39–51 of Arp3, residues 288–297 and 309–319 of ARPC1, and 41–43 and 65–67 of the Arp2 model. All phosphorylated and mutant models were generated from the unphosphorylated model by removing all side chain atoms from the unmodified residue and optimizing the positions of the side-chain atoms of the modified residue. These models were then solvated in TIP3P water [50] and monovalent counterions were added to neutralize the system using Maestro (Schrodinger LLC).

The full system was then energy minimized using DESMOND [51] (D.E. Shaw Research) in five stages with the following atoms restrained to their positions in the starting model: 1) all heavy atoms; 2) all backbone (N-C〈-C-O) heavy atoms and experimentally determined side-chain heavy atoms; 3) all experimentally determined heavy atoms; 4) all experimentally determined backbone atoms; 5) no restraints. Minimizations were performed with at least 100 steps of Steepest Descent minimization followed by L-BFGS optimization after reaching a gradient of 10.0 kcal·mol^−1^·Å^−1^ up to a total of 10,000 steps or a gradient of 0.1 kcal·mol^−1^·Å^−1^. After full energy minimization of the system, an equilibration was performed. First, the systems were annealed to a temperature of 300 K using Langevin dynamics at constant temperature and volume over 50 ps with all heavy atoms restrained. Subsequently, Langevin dynamics at constant temperature and pressure with a target temperature and pressure of 300 K and 1 atm were performed in stages: 1) 50 ps with all heavy atoms restrained with 50 kcal·mol^−1^·Å^−1^ force constants; 2) 50 ps with all backbone heavy atoms and experimentally determined side-chain atoms restrained with 50 kcal·mol^−1^·Å^−1^ force constants; 3) 150 ps with all experimentally-determined heavy atoms restrained with force constants reduced over the course of the simulation from 25 to 5 kcal·mol^−1^·Å^−1^; 4) 100 ps of simulation restraining only the experimentally determined backbone heavy atoms, over which the force constants of the restraints were brought to 0 from 5.0 kcal·mol^−1^·Å^−1^; 5) 100 ps of the unrestrained system. All Langevin dynamics simulations were performed with a 100 ps^−1^ damping constant.

Each system was then simulated for 30 ns using the Martyna-Tobias-Klein integrator [52] with a reference temperature of 300 K and a reference pressure of 1 atm. The barostat mass was set with a time constant of 2 ps and an equilibrium temperature of 300 K. The masses of all chain variables were set using a time constant of 1.0 ps. Both the Langevin dynamics and standard molecular dynamics simulations were performed with all bonds involving hydrogens constrained, a 2 fs time step for the bonded and short-range nonbonded interactions and updating of long-range nonbonded interactions every 4 fs using the RESPA multiple time step approach. Non-bonded interactions were tapered using force-switching starting at a distance of 9.0 Å to an interaction cutoff of 9.5 Å. Pairlists were constructed using a distance of 10.5 Å and a migration interval of 12 ps. These parameters were tested in short simulations in the NVE ensemble to ensure good energy conservation. Coordinates of the full system were added to the output trajectory every 10 ps.

### Principal component analysis

Coordinates of the Cα atoms from the last 20 ns of each unphosphorylated, Arp2 pThr237, and Arp2 pThr238 simulation were collected into a single trajectory on which Principal Component Analysis [53], [54] was performed using the Bio3D package for the R statistical software package [55]. All Cα coordinates were used after superimposing the Cα atoms of Arp3 subdomains 1 and 2 resolved in the starting crystal structure (residues 3–39, 51–151, 376–410) of each frame in each trajectory. The first and second principal components (PCs) account for 62.4% of the variation in atomic coordinates, and the first 4 principal components account for 84.0% (**Fig. S3a**). The major differences between the unphosphorylated and phosphorylated simulations are largely localized to the first PC, with the second PC capturing variation between the duplicate simulations of the complex in the same phosphorylation state.

### Calculation of number of Arp2-Arp3 contacts

A contact between the Arp2 and Arp3 subunits was defined as the number of heavy atoms in Arp3 that were within 3.5 Å of any heavy atom in Arp2. The number of contacts between the Arp2 and Arp3 subunits was calculated for every 10^th^ frame (100 ps) of each simulation. The results for the duplicate simulations of each wild-type or mutant complex were then pooled and compared.

## Supporting Information

Figure S1 **NPF and F-actin binding affinities were measured by pelleting assays with rArp2/3 complex.** (a) Arp2/3 binding to NPF was similar for WT, T237/238A-Y202A Arp2, R105/106A ARPC1 Arp2/3 complex (b) Arp2/3 binding to filamentous actin was similar for WT, T237/238A-Y202A Arp2, R105/106A ARPC1 Arp2/3 complex.
(TIFF)

Figure S2 **Creation of new filament barbed ends upon Arp2/3 complex activation.** (a) Concentrations of filaments from pyrene actin assembly assays plotted as a function of Arp2/3 concentrations comparing native and recombinant WT Arp2/3 complex either untreated or treated with Antarctic Phosphatase. (b) Concentrations of filaments from pyrene actin assembly assays plotted as a function of Arp2/3 concentrations comparing WT and recombinant T237/238A-Y202A Arp2 Arp2/3 complex either untreated or treated with Antarctic Phosphatase.
(TIFF)

Figure S3 **Principal component analysis of dominant differences between unphosphorylated and Arp2 T237 or T238 phosphorylated Arp2/3 complex.** (a) Scree plot showing the proportion of variance in atomic displacement accounted for by each principal component (PC), sorted from highest to lowest eigenvalue. The total proportion of variance accounted for by all PCs with equal or greater eigenvalue than a given PC are indicated next to points on the plot. (b) Snapshots from the last 20 ns of the duplicate unphosphorylated simulations (black), Arp2 T237 phosphorylated simulations (slate blue), and Arp2 T238 phosphorylated simulations (turquoise) were projected onto the first and second principal components describing the variation in atomic displacements. Discrimination of unphosphorylated and phosphorylated states is achieved along the first principal component, while the second principal component exhibits variation between the two independent simulations in each phosphorylation state. The projection of the starting crystal structure (PDB 1K8K [25]) on this set of principal components is shown as a red triangle. (c) Porcupine plot of the first principal component showing large subunit rearrangements of Arp2, ARPC1, and ARPC3 relative to the rest of the complex. The Cα coordinates displaced by one standard deviation of the conformer distribution from the average structure (of the unphosphorylated, Arp2 pT237, and Arp2 pT238 simulations) along the positive direction of PC1 are shown as a chain trace, and cones are drawn to the Cα coordinates displaced by one standard deviation of the conformer distribution in the negative direction of PC1. Since the states sampled by the phosphorylated simulations sample more negative values of PC1, the cones reflect the direction and relative size of atomic displacements needed to progress from the unphosphorylated structural states to the phosphorylated structural states. This image was produced using the molecular graphics program VMD [57]. Principal component analysis was performed using the Bio3D package [55] for the R statistical software program.
(TIFF)

Figure S4 **Arp2-Arp3 orientation of starting model and phosphorylated Arp2 T238 compared with the unphosphorylated simulation.** Stereoimages of Arp3 and Arp2 subunits from a snapshot from the last ns of the unphosphorylated (Arp3-gray; Arp2-blue) simulation compared with (a) starting model of unphosphorylated Arp2/3 complex based on the crystal structure by Robinson, et al [25] (pink, purple) and (b) a snapshot from the last ns of phosphorylated T238 Arp2 (yellow, green) wild-type simulations are shown following alignment of Cα atoms of subdomains 1 and 2 of Arp3. The other subunits of the complex from the snapshot of the unphosphorylated simulation, colored as in Fig. 2a, were represented as transparent surfaces in order not to occlude the views of Arp2 and Arp3.
(TIFF)

Figure S5 **Arp2 average RMSD by residue for wild-type simulations.** The per-residue Cα RMSD averaged over the last 20 ns after alignment of (a) Arp3 subdomains 1 and 2 or b) Arp2 backbone atoms. The colors are as follows: U(black)- unphosphorylated; pT237(blue-gray) – phosphorylated T237 Arp2; pT238(cyan) – phosphorylated T238 Arp2.
(TIFF)

Figure S6 **Interactions in the vicinity of the T238 Arp2 phosphorylation site.** Stereoimages of the salt-bridge network between Arp2 (pink), ARPC4 (green), and Arp3 (blue) in the (a) unphosphorylated and the (b) phosphorylated T238 Arp2 simulations, focused on the vicinity of T238. Portions of the structure have been removed for clarity, including the backbones of Arp2 residues K253 and R200.
(TIFF)

Figure S7 **Distribution of hydrogen bond donor-acceptor distances in the vicinity of the T237 and T238 Arp2 phosphorylation sites.** The distribution of minimum distances between hydrogen bond donor and acceptor distances over the last 20 ns of unphosphorylated (black), T237 Arp2 phosphorylated (blue-gray), and T238 Arp2 phosphorylated (cyan) simulations. (a)–(g) show interactions in the vicinity of the T237 phosphorylation site, and (h)–(l) show interactions in the vicinity of the T238 phosphorylation site. In (f), the black line for the unphosphorylated simulation superimposes with the cyan line for the phosphorylated T238 Arp2 simulation. Please note that the scales on each plot vary to clearly show similarities and differences within each independent set of distributions.
(TIFF)

Figure S8 **Comparison of actin filament end concentrations as a function of Arp2/3 concentration.** The plot compares filament end concentrations of WT versus R105/106A ARPC4 Arp2/3 complex either untreated or treated with Antarctic Phosphatase in the absence or presence of N-WASP VCA.
(TIFF)

Table S1 **Affinity of Arp2/3 complexes to actin filament pointed ends.** The Arp2/3 complexes and their measured affinities to actin filament pointed ends are shown for: wild-type recombinant Arp2/3 complex (WT rArp2/3); Antarctic phosphatase treated wild-type recombinant Arp2/3 complex (AP-WT rArp2/3); recombinant Arp2/3 complex with Arp2 T237A, T238A, and Y202A mutations (T237/T238 Y202A Arp2/3); recombinant Arp2/3 complex with ARPC4 R105A and R106A mutations (R105/106A ARPC4 Arp2/3); and Antarctic phosphatase treated recombinant Arp2/3 complex with ARPC4 R105A and R106A mutations (AP-R105/106A ARPC4 Arp2/3).
(DOC)
