# Supplementary figures and images for: Phosphorylation of the Arp2 Subunit Relieves Auto-inhibitory Interactions for Arp2/3 Complex Activation
Source: PLoS Comput Biol. 2011 Nov 10;7(11):e1002226. doi: 10.1371/journal.pcbi.1002226 (PMC3220268; doi:10.1371/journal.pcbi.1002226)

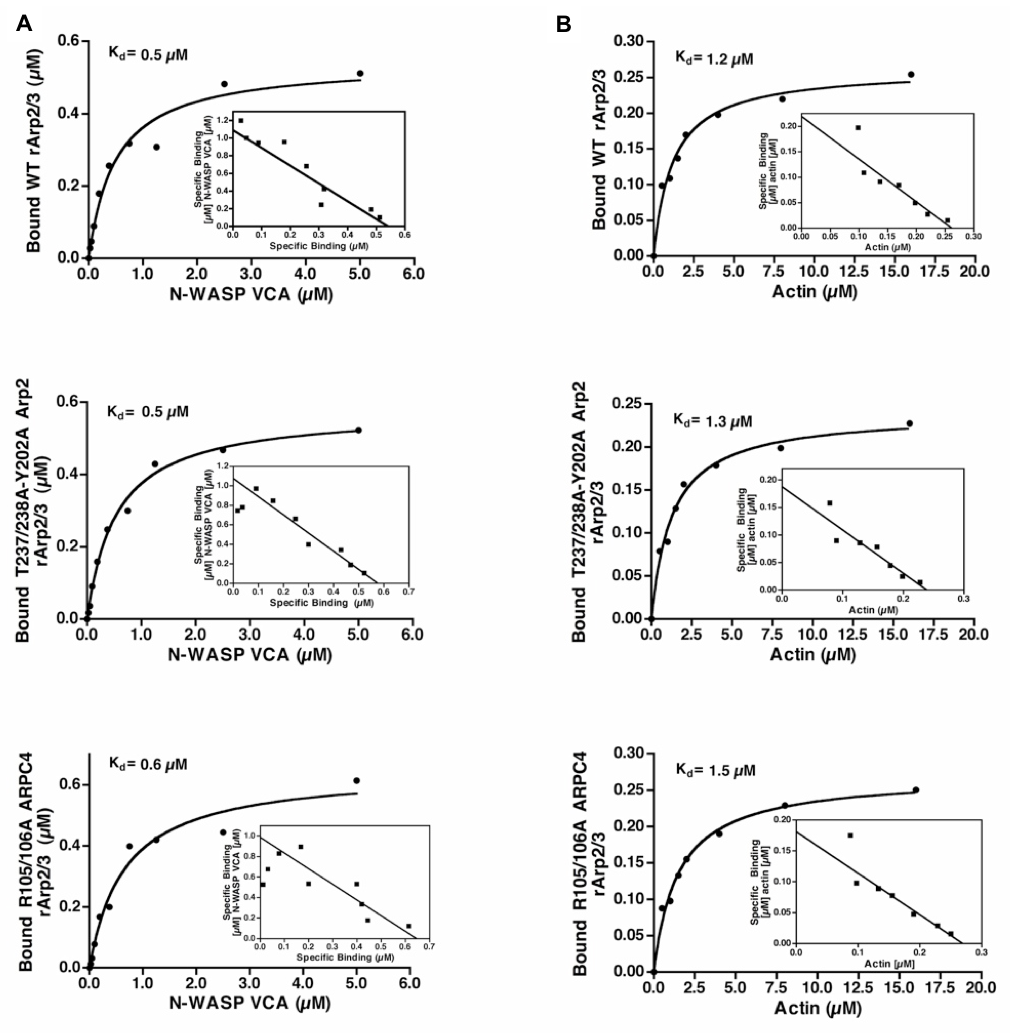

Supplement: Figure S1 — NPF and F-actin binding affinities were measured by pelleting assays with rArp2/3 complex. (a) Arp2/3 binding to NPF was similar for WT, T237/238A-Y202A Arp2, R105/106A ARPC1 Arp2/3 complex (b) Arp2/3 binding to filamentous actin was similar for WT, T237/238A-Y202A Arp2, R105/106A ARPC1 Arp2/3 complex. (TIFF) [file pcbi.1002226.s001.tif]

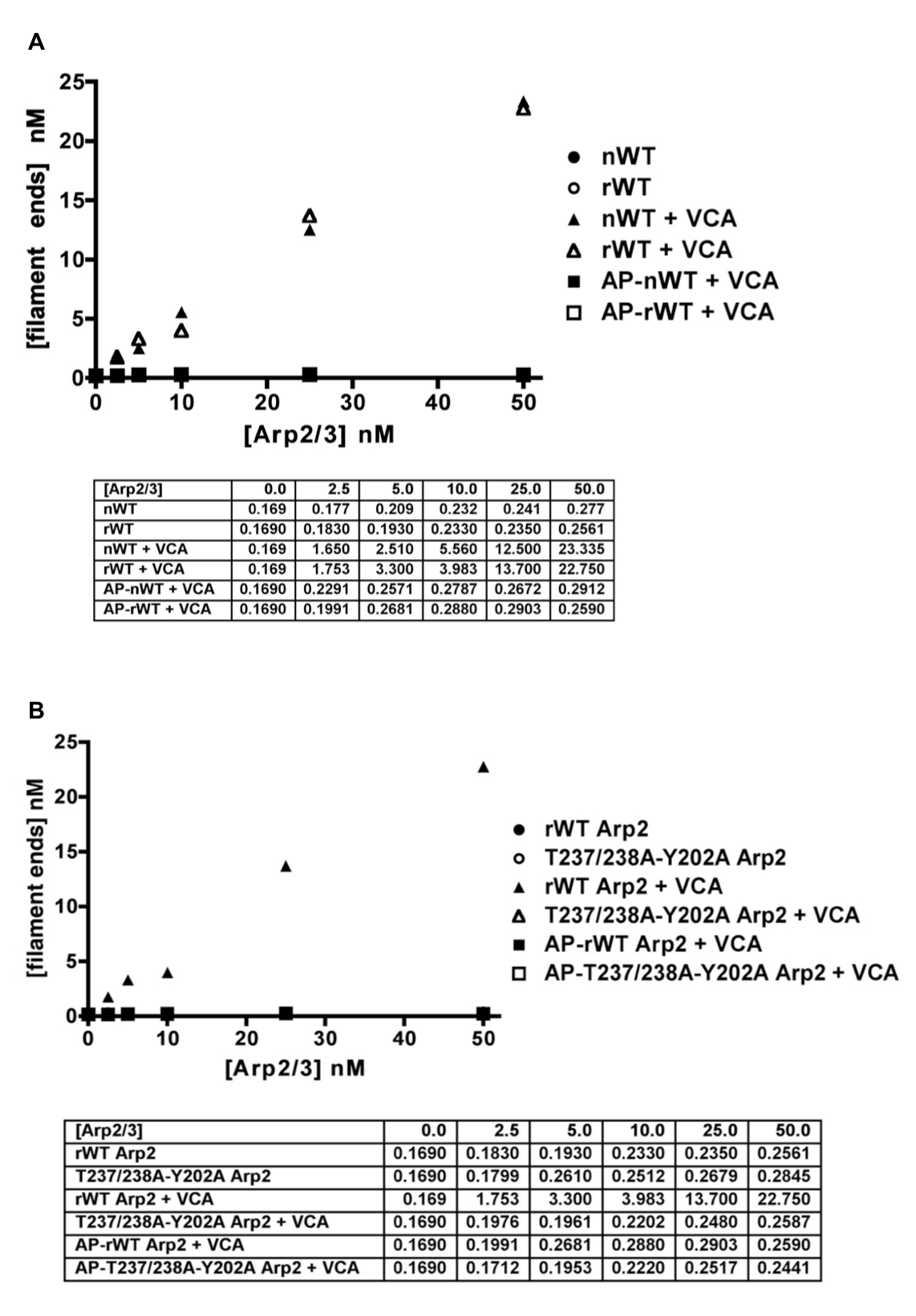

Supplement: Figure S2 — Creation of new filament barbed ends upon Arp2/3 complex activation. (a) Concentrations of filaments from pyrene actin assembly assays plotted as a function of Arp2/3 concentrations comparing native and recombinant WT Arp2/3 complex either untreated or treated with Antarctic Phosphatase. (b) Concentrations of filaments from pyrene actin assembly assays plotted as a function of Arp2/3 concentrations comparing WT and recombinant T237/238A-Y202A Arp2 Arp2/3 complex either untreated or treated with Antarctic Phosphatase. (TIFF) [file pcbi.1002226.s002.tif]

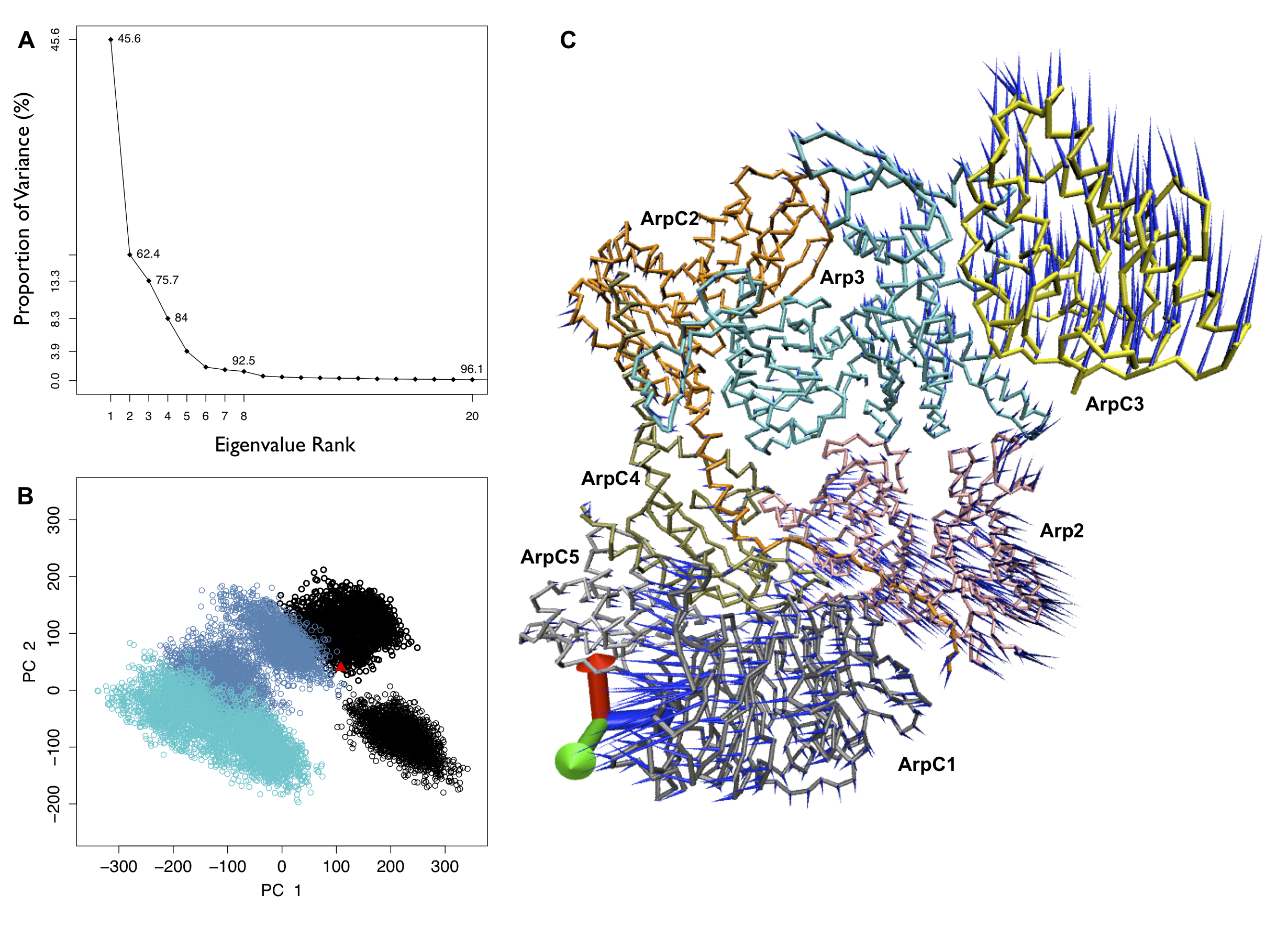

Supplement: Figure S3 — Principal component analysis of dominant differences between unphosphorylated and Arp2 T237 or T238 phosphorylated Arp2/3 complex. (a) Scree plot showing the proportion of variance in atomic displacement accounted for by each principal component (PC), sorted from highest to lowest eigenvalue. The total proportion of variance accounted for by all PCs with equal or greater eigenvalue than a given PC are indicated next to points on the plot. (b) Snapshots from the last 20 ns of the duplicate unphosphorylated simulations (black), Arp2 T237 phosphorylated simulations (slate blue), and Arp2 T238 phosphorylated simulations (turquoise) were projected onto the first and second principal components describing the variation in atomic displacements. Discrimination of unphosphorylated and phosphorylated states is achieved along the first principal component, while the second principal component exhibits variation between the two independent simulations in each phosphorylation state. The projection of the starting crystal structure (PDB 1K8K [25]) on this set of principal components is shown as a red triangle. (c) Porcupine plot of the first principal component showing large subunit rearrangements of Arp2, ARPC1, and ARPC3 relative to the rest of the complex. The Cα coordinates displaced by one standard deviation of the conformer distribution from the average structure (of the unphosphorylated, Arp2 pT237, and Arp2 pT238 simulations) along the positive direction of PC1 are shown as a chain trace, and cones are drawn to the Cα coordinates displaced by one standard deviation of the conformer distribution in the negative direction of PC1. Since the states sampled by the phosphorylated simulations sample more negative values of PC1, the cones reflect the direction and relative size of atomic displacements needed to progress from the unphosphorylated structural states to the phosphorylated structural states. This image was produced using the molecular graphics program VMD [57]. Princi [file pcbi.1002226.s003.tif]

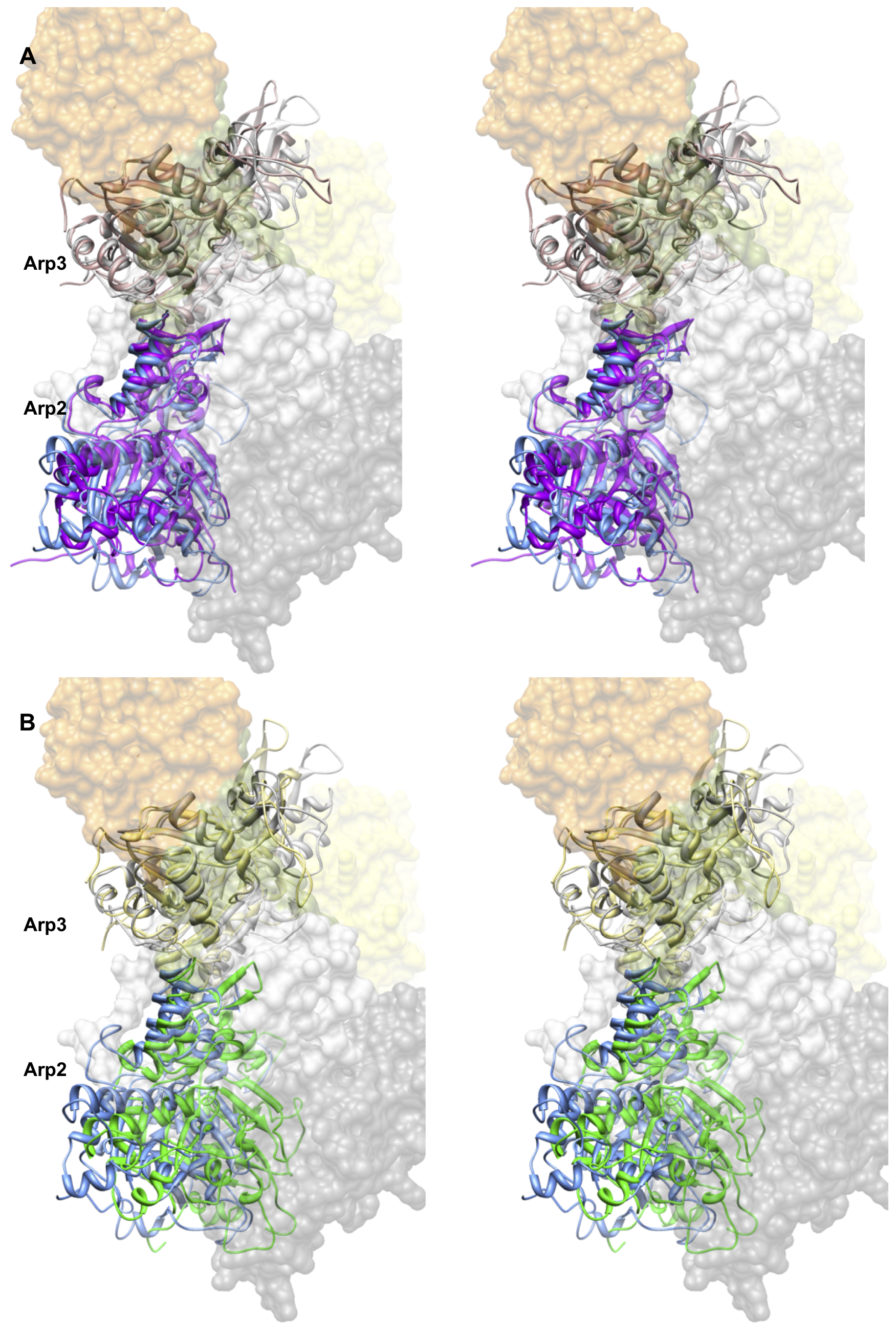

Supplement: Figure S4 — Arp2-Arp3 orientation of starting model and phosphorylated Arp2 T238 compared with the unphosphorylated simulation. Stereoimages of Arp3 and Arp2 subunits from a snapshot from the last ns of the unphosphorylated (Arp3-gray; Arp2-blue) simulation compared with (a) starting model of unphosphorylated Arp2/3 complex based on the crystal structure by Robinson, et al [25] (pink, purple) and (b) a snapshot from the last ns of phosphorylated T238 Arp2 (yellow, green) wild-type simulations are shown following alignment of Cα atoms of subdomains 1 and 2 of Arp3. The other subunits of the complex from the snapshot of the unphosphorylated simulation, colored as in Fig. 2a, were represented as transparent surfaces in order not to occlude the views of Arp2 and Arp3. (TIFF) [file pcbi.1002226.s004.tif]

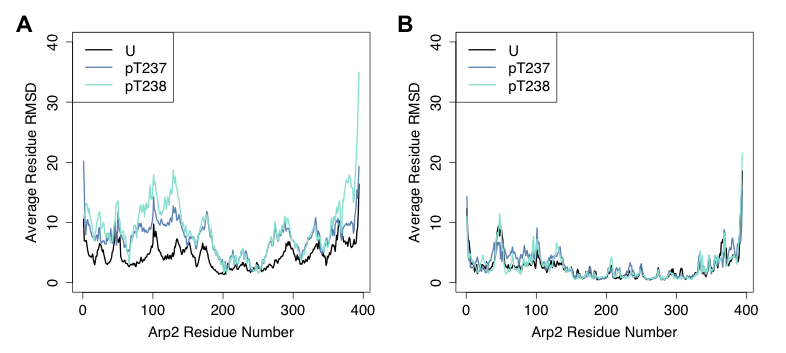

Supplement: Figure S5 — Arp2 average RMSD by residue for wild-type simulations. The per-residue Cα RMSD averaged over the last 20 ns after alignment of (a) Arp3 subdomains 1 and 2 or b) Arp2 backbone atoms. The colors are as follows: U(black)- unphosphorylated; pT237(blue-gray) – phosphorylated T237 Arp2; pT238(cyan) – phosphorylated T238 Arp2. (TIFF) [file pcbi.1002226.s005.tif]

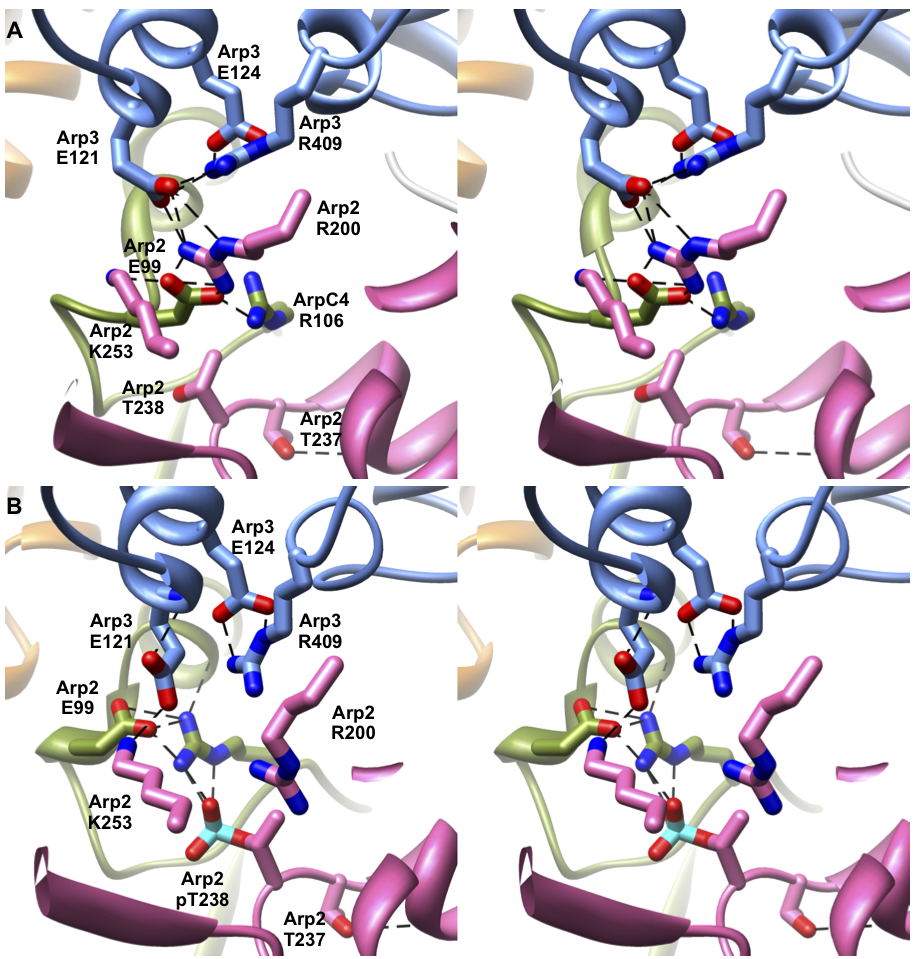

Supplement: Figure S6 — Interactions in the vicinity of the T238 Arp2 phosphorylation site. Stereoimages of the salt-bridge network between Arp2 (pink), ARPC4 (green), and Arp3 (blue) in the (a) unphosphorylated and the (b) phosphorylated T238 Arp2 simulations, focused on the vicinity of T238. Portions of the structure have been removed for clarity, including the backbones of Arp2 residues K253 and R200. (TIFF) [file pcbi.1002226.s006.tif]

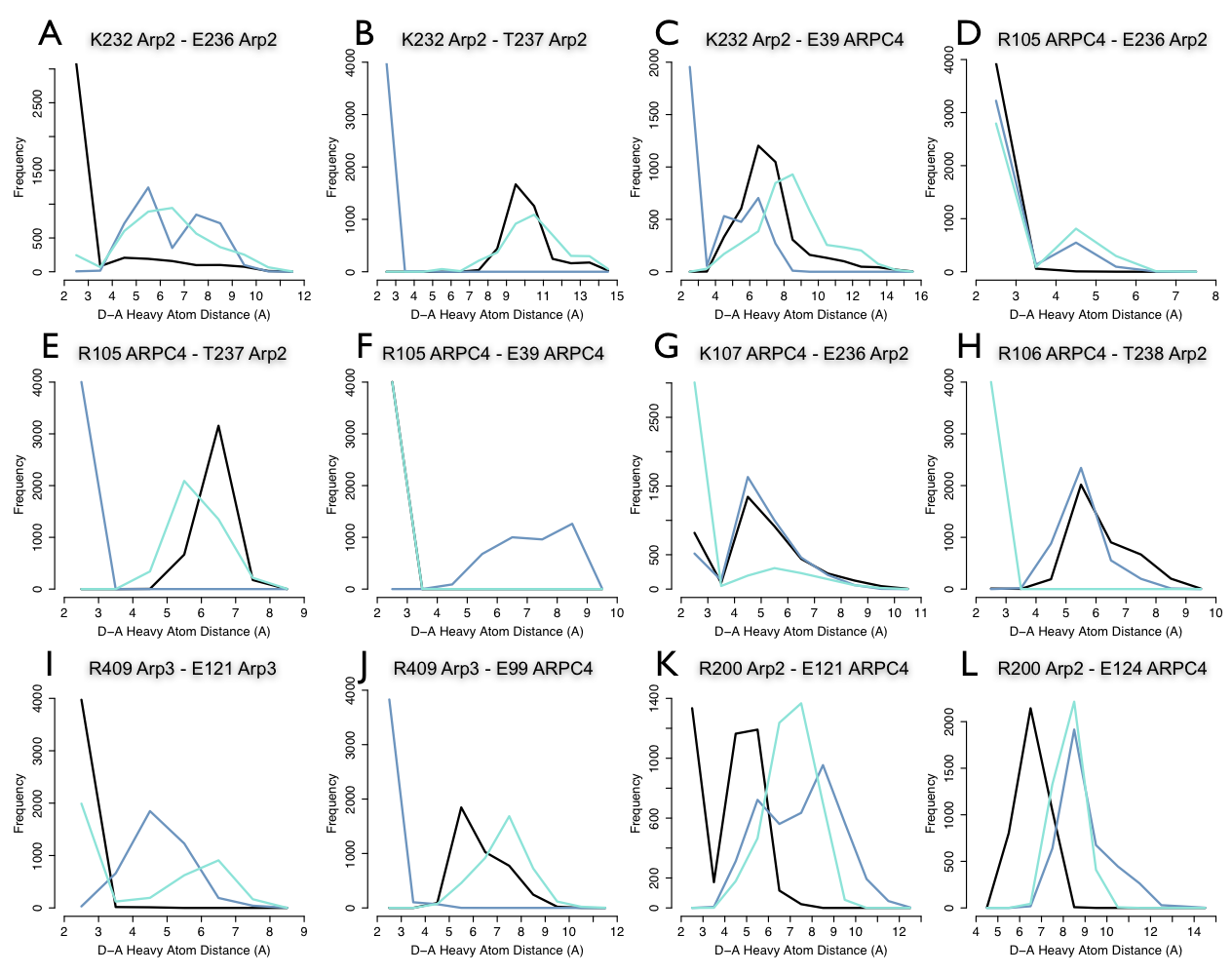

Supplement: Figure S7 — Distribution of hydrogen bond donor-acceptor distances in the vicinity of the T237 and T238 Arp2 phosphorylation sites. The distribution of minimum distances between hydrogen bond donor and acceptor distances over the last 20 ns of unphosphorylated (black), T237 Arp2 phosphorylated (blue-gray), and T238 Arp2 phosphorylated (cyan) simulations. (a)–(g) show interactions in the vicinity of the T237 phosphorylation site, and (h)–(l) show interactions in the vicinity of the T238 phosphorylation site. In (f), the black line for the unphosphorylated simulation superimposes with the cyan line for the phosphorylated T238 Arp2 simulation. Please note that the scales on each plot vary to clearly show similarities and differences within each independent set of distributions. (TIFF) [file pcbi.1002226.s007.tif]

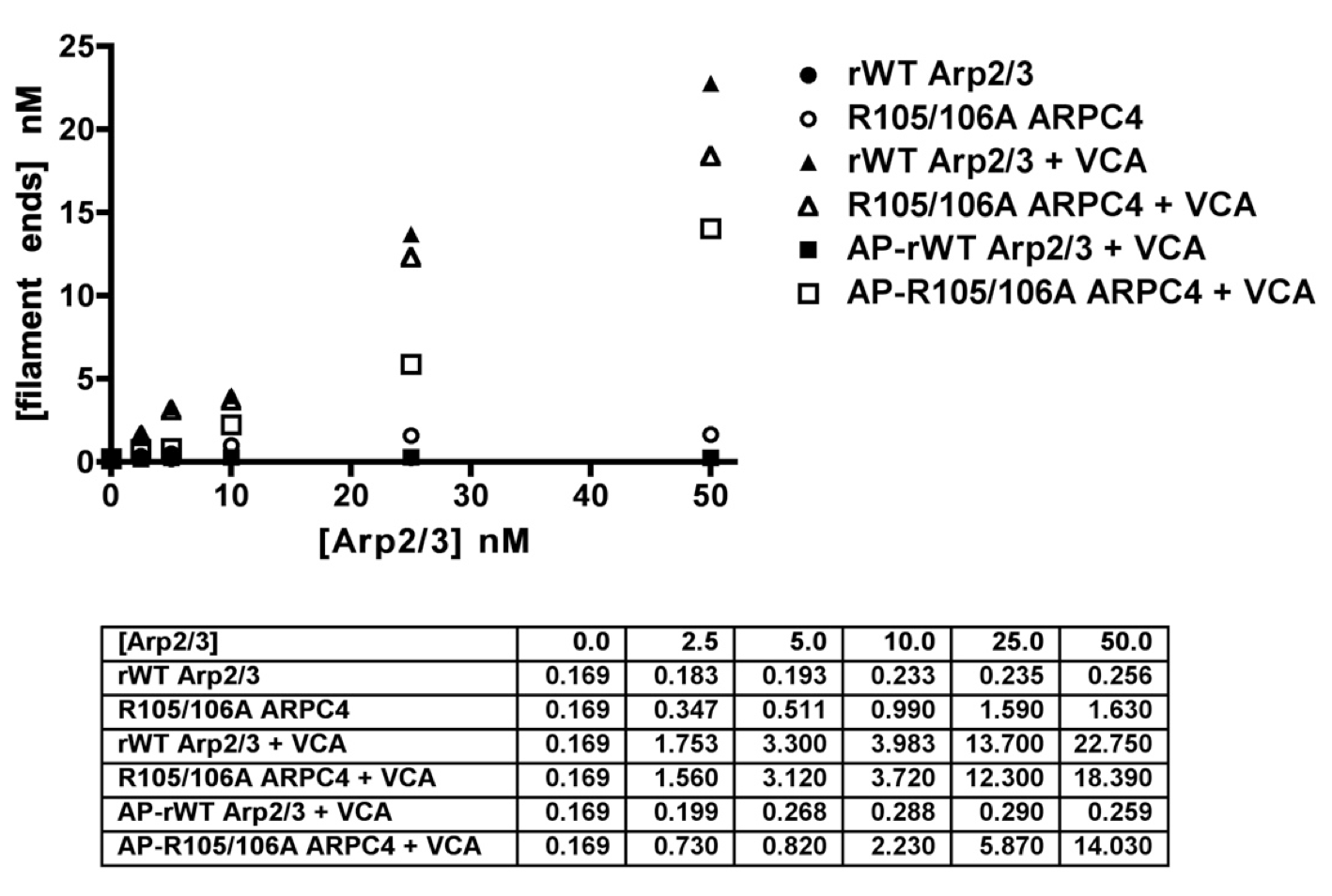

Supplement: Figure S8 — Comparison of actin filament end concentrations as a function of Arp2/3 concentration. The plot compares filament end concentrations of WT versus R105/106A ARPC4 Arp2/3 complex either untreated or treated with Antarctic Phosphatase in the absence or presence of N-WASP VCA. (TIFF) [file pcbi.1002226.s008.tif]
