# Supplementary material for: Phosphorylation of the Arp2 Subunit Relieves Auto-inhibitory Interactions for Arp2/3 Complex Activation
Source: PLoS Comput Biol. 2011 Nov 10;7(11):e1002226. doi: 10.1371/journal.pcbi.1002226 (PMC3220268; doi:10.1371/journal.pcbi.1002226)
Supplement: Table S1 — Affinity of Arp2/3 complexes to actin filament pointed ends. The Arp2/3 complexes and their measured affinities to actin filament pointed ends are shown for: wild-type recombinant Arp2/3 complex (WT rArp2/3); Antarctic phosphatase treated wild-type recombinant Arp2/3 complex (AP-WT rArp2/3); recombinant Arp2/3 complex with Arp2 T237A, T238A, and Y202A mutations (T237/T238 Y202A Arp2/3); recombinant Arp2/3 complex with ARPC4 R105A and R106A mutations (R105/106A ARPC4 Arp2/3); and Antarctic phosphatase treated recombinant Arp2/3 complex with ARPC4 R105A and R106A mutations (AP-R105/106A ARPC4 Arp2/3). (DOC) [file pcbi.1002226.s009.doc]

**Supplementary Table** 1

| **Arp2/3** | **K_d_ (SD)** |
| --- | --- |
| WT rArp2/3 | 77 ± 23 nM |
| AP-WT rArp/2 | 758 ± 224 nM |
| T237/238 Y202A Arp2/3 | 601 ± 173 nM |
| R105/106A ARPC4 Arp2/3 | 120 ± 37 nM |
| AP-R105/106A ARPC4 Arp2/3 | 205 ± 82 nM |
